# Aptamer based electrochemical sensors for emerging environmental pollutants

**DOI:** 10.3389/fchem.2014.00041

**Published:** 2014-06-26

**Authors:** Akhtar Hayat, Jean L. Marty

**Affiliations:** ^1^BIOMEM, Université de PerpignanPerpignan, France; ^2^Interdisciplinary Research Centre in Biomedical Materials, COMSATS Institute of Information TechnologyLahore, Pakistan

**Keywords:** electrochemical sensor, aptamer, emerging pollutants, environmental applications, analytical monitoring

## Abstract

Environmental contaminants monitoring is one of the key issues in understanding and managing hazards to human health and ecosystems. In this context, aptamer based electrochemical sensors have achieved intense significance because of their capability to resolve a potentially large number of problems and challenges in environmental contamination. An aptasensor is a compact analytical device incorporating an aptamer (oligonulceotide) as the sensing element either integrated within or intimately associated with a physiochemical transducer surface. Nucleic acid is well known for the function of carrying and passing genetic information, however, it has found a key role in analytical monitoring during recent years. Aptamer based sensors represent a novelty in environmental analytical science and there are great expectations for their promising performance as alternative to conventional analytical tools. This review paper focuses on the recent advances in the development of aptamer based electrochemical sensors for environmental applications with special emphasis on emerging pollutants.

## Introduction

Nucleic acid, as the biological polymer for the storage and propagation of genetic information, possess remarkable structural and functional characteristics (Tang et al., 2014). Highly specific base pairing properties of nucleic acid permit for replication and transcription processes (Seeman, 2003). Recently, nucleic acid has been emerged as a powerful and versatile building block in the construction of biosensors (Seeman, 2010). A variety of nucleic acid (DNA or RNA) based biosensors have been designed and reported in the literature. Among them, aptamers are nucleic acid (DNA or RNA) that selectively bind to low molecular weight organic or inorganic substrate or to relatively big macromolecules. The affinity constant of aptamers toward their specific targets is reported in the micromolar to pico molar ranges, comparable to the binding constant of antibody and antigen affinity interaction (Jenison et al., 1994; Willner and Zayats, 2007). They are engineered through an *in vitro* selection procedure, also called SELEX (Systematic Evolution of Ligands by EXponential enrichment), which was first reported in 1990 (Hayat et al., 2012b, 2013c; Paniel et al., 2013).

Recently, aptamers have found tremendous interest as an active separation material in chromatography and electrophoresis, therapeutic and diagnostic agent, and as acting recognition material in biosensing to replace the commonly used bioreceptors (Ellington and Szostak, 1990; Hermann and Patel, 2000; Hamula et al., 2006; Tan et al., 2013). Aptamers offer many advantages compared to antibodies, which are biologically produced antigen specific proteins. The production of aptamer does not require an immune response in host animals to obtain them, as they are chemically produced by automated nucleic acid synthesis. Similarly, the antibodies cannot be easily obtained for small size targets (e.g., metal ions) or for molecules with poor immunogenicity or high toxicity, while there is a possibility to design aptamer against such target analytes. Besides, aptamers can be very easily chemically modified which permits to immobilize them over wide range of transducer surfaces (O'Sullivan, 2002; Gorodetsky et al., 2008). Moreover, the properties of conformational changes upon target-analyte binding make them most appropriate and suitable candidate to design label free and portable biodevices for analytical applications. This conformational alteration characteristic of aptamer facilitates and enhances the detection phenomena of small size target analytes by enfolding them in the folded DNA structures. For large molecules such as proteins, the folded DNA aptamer bind to a particular epitope. In principle, aptamer based biosensors can be fabricated to respond to any ligand for which an aptamer exists (Wang et al., 2011; Tang et al., 2014). They are widely regarded as ideal recognition element for diverse analytical applications, particularly environmental analysis.

Recent years have witnessed increasing need to monitor the environmental contaminations. Food, air and water are the main victims of the contaminants that may have impact on human and animal life. The environmental contaminants have mild to severe short-term or long term effect and some of them even have deadly effects and lead to widespread havoc. The contaminants that need monitoring in the environment can be broadly classified as small organic and inorganic pollutants, pharmaceutical and personal care products, toxins of microbial origin and pathogens. Although there has been lot of interest in developing techniques for monitoring of environmental pollutants, there is still great demand for portable, decentralized and highly robust assays (Cella et al., 2011). Chromatographic methods are the traditionally used assays for quantitative and qualitative measurement of environmental pollutants. Although these methods are very sensitive and selective, but they still require costly instruments and trained person to perform the analysis, in addition to being unsuitable for decentralized analysis. Biosensors based on the antibody as bio-recognition element have been emerged for environmental monitoring. Because of the expensive animal models required to produce antibody, unavailability against nonimmunogenic contaminants and instability under varying physiological conditions, antibodies are not potential candidates for environmental monitoring analysis. Alternatively, RNA or DNA Aptamers have attained great attraction in the field of environmental monitoring. Apart from having the same or even higher sensitivity and selectivity as antibodies, aptamers offer the advantages of large scale production with less expensive *in vitro* system and enhanced environmental stability. Aptamers due to their ease of modification with various functional groups can be integrated into electrochemical biosensing platform. This review summarizes the accomplishment, and highlights the advantages of electrochemical aptasensors for environmental samples analysis.

## Electrochemical signaling of aptamer constructs

Transduction of the affinity binding event to measurable signal is usually obtained through optical output in aptamer based assays. Traditionally optical based read out methods of aptamer binding event not only require high precise and expensive instrumentation but also involve sophisticated numerical algorithms to interpret the data. Alternatively, a number of innovative designs of electrochemical aptasensors have been reported in the literature. This type of devices combined aptamer with electrochemical transducers to generate an electrical signal, and provides a simple, accurate and an inexpensive platform for applications such as environmental monitoring.

### Advantages of electrochemical methods

Among all the transduction approaches, electrochemical detection is an attractive sensing platform in the field of biosensors (Barthelmebs et al., 2011; Hayat et al., 2011, 2012a). It was not explored in aptasensing until 7 years ago; however, since these last years, electrochemical transduction has received much attention to monitor the behavior of target-aptamer complex on electrode surface (Hayat et al., 2013a,b). An electrochemical device measures change in the electric current produced by redox reactions occurring on the transducer electrode surface (Hayat and Andreescu, 2013). This sensing methodology has various advantages including simplicity, rapidity, low cost and high sensitivity. The use of aptamer as bio-recognition element is strongly favored for some electrochemical detection techniques. Till now, plenty of research work has been devoted to the development of electrochemical aptasensors. Electrochemical aptasensor for biomolecular reaction monitoring is mainly based on the detection of current or potential changes resulting from interactions occurring at the transducer surface. It presents several advantages over optical, piezoelectric or thermal detection. Electrochemical transduction is simple, rapid, cost effective, highly sensitive and selective, compatible with novel micro-fabrication techniques, disposable, easy to miniaturize, robust, and independent of sample turbidity, in view of high-throughput. Electrochemical reactions usually provide an electronic signal directly, avoiding the requirement of expensive signal transduction equipment (Castillo et al., 2012; Rhouati et al., 2013a,b).

**Figure 1:**
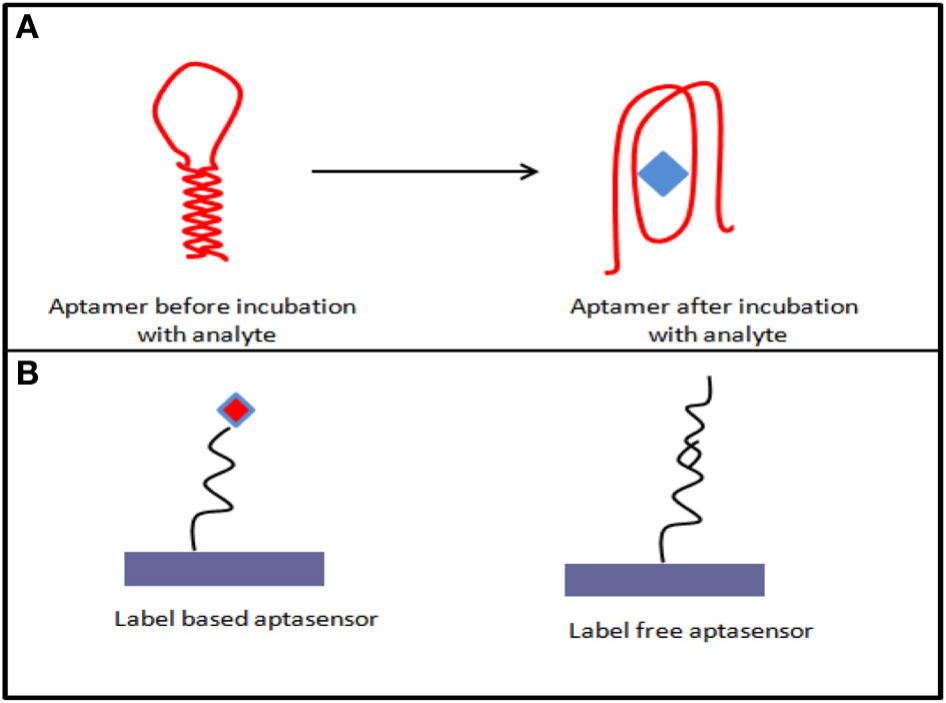
**(A)** Aptamer conformational changes after target analyte incubation; **(B)** various possible formats of aptasensing.

As aptamer are nucleic acid strand and can be very easily immobilized on the transducer electrode surface, chip-based, portable, and miniaturized electrochemical systems can be designed accordingly. For electrochemical aptasensor, most of the commonly used electrode materials are gold and carbon surfaces on which aptasensor construct can be built via different immobilization strategies and detection formats (Figure 1) (Lai et al., 2006; White et al., 2008).

### Conventional methods for electrochemical aptasensing

**Figure 2:**
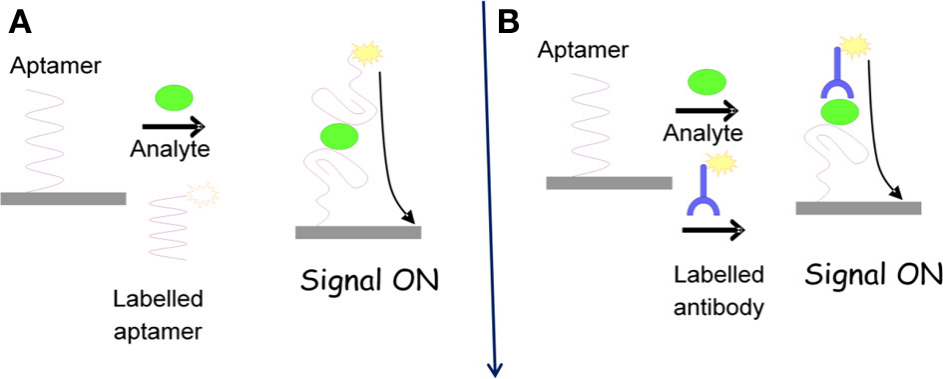
**Signal ON formats for aptasensing**.

**Figure 3:**
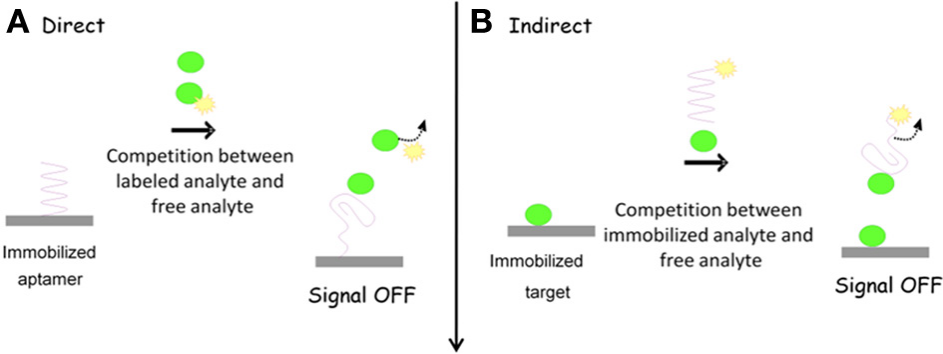
**Signal OFF formats for aptasensing**.

There is a great variety of different labels which have been applied in electrochemical aptasensors. These reporters can be covalently conjugated to the aptamer itself, conjugated to a complementary oligonucleotide, or indirectly attached with aptamers. Following the incorporation of an electrochemical reporter, detection can be either signal-on (Figure 2) or signal-off (Figure 3), depending on the format of the assay. Among the most valuable labels are enzymes such as horse radish peroxidase (HRP), glucose oxidase (GOD), alkaline phosphatase (ALP). Other reporters can also be used to overcome problems typically associated with enzymes. In particular, electroactive compounds such as ferrocene, ferrocyanide, methylene blue (MB), Pt and Cds quantum dots (QDs), and other nanoparticles (NPs) offer a number of advantages over standard enzymes for monitoring biological systems (Ikebukuro et al., 2005; Mir et al., 2006; Centi et al., 2007; Park et al., 2011).

### Redox labeling methods for aptasensors

Sensitive electrochemical signaling is usually based on the redox properties of the reported molecules confined to the transducer surface. Different redox labels for electrochemical aptasensor have been developed in the past two decade. Various reporter molecules including ferrocene and mthylene blue have been covalently tethered to the distal terminus of aptamer through flexible alkyle linkages. Based on the restriction of redox reporter mobility, the position of the aptamer can be controlled by varying their orientation or surface density. When the length of alkyle linkages is limited, the reaction is only a surface controlled process, without involvement of diffusion processes resulting in simplified electrochemical output data (Ihara et al., 1996; Pheeney and Barton, 2012). Intercalation is another procedure to insert a ligand between the base pair of DNA through non covalent interactions. The ligands called intercalators mostly include ploycyclic, aromatic, and planar molecule (Kelley et al., 1997, 1999). Similarly, negatively charged reports which are freely diffusing in solution, cannot get into close proximity with the aptmaer modified transducer surface due to electrostatic interactions. For example ferri/ferrocynide ions in the solution are repelled by the negatively charged species, increasing the electron transfer resistance. This increase in electron transfer resistance can be measured as a function of analyte concentration by using electrochemical impedance spectroscopy (Yu et al., 2003; Cheng et al., 2007).

### Rational design of electrochemical aptasensors

In functional aptamer based electrochemical sensors, aptamer undergoes various structural or conformational changes in the presence of external stimuli, resulting in an alteration in the electrochemical signal. The most commonly explored pattern in this context is aptamer-ligand binding phenomena. When aptamers are immobilized on the electrode surface, the binding of the target analyte may induce structural and conformational changes. In the presence of redox reported, electrochemical signals can be obtained by employing conventional electrochemical techniques. Aptmaer constructs are mainly single or double stranded in which the target binding induces the dissociation of duplexes or the folding of single strands. Aptamers may bind to their target analytes by two different recognition events; (a) binding to their ligands and (b) binding to their Watson-Crick complementary strand. This competition can be employed to design novel types of electrochemical aptasensors. These types of formats form a fully or partially double stranded DNA sensor with one strand incorporating aptamer sequence. Upon incubation with target analyte, the aptamer selectively binds to the analyte with the dissociation of the DNA duplex, which is further employed to get the electrochemical signal (Zuo et al., 2007; Lu et al., 2008; Chakraborty et al., 2009). Alternatively, folding of single strand aptmaer is used to construct the electrochemical sensors. This type of aptasensor design offer advantages such as; (a) the aptamer conformational changes upon target analyte bindings are simple and does not involve the dissociation of the duplex, (b) surface can be very easily regenerated, without requirement of re-hybridization of the dissociated strand. The later type of format is mostly commonly used to fabricate simple and robust electrochemical aptasensors (Xiao et al., 2005; Baker et al., 2006; Radi et al., 2006).

## Different methods of aptamer immobilization

Immobilization of aptamer on the transducer surface is one of the fundamental and critical steps in the design of electrochemical aptasensors. Aptamer can be immobilized on electrode surface via 5′-end or the 3′-end. However, the 3′ end is more suitable because it simultaneously confers resistance to nuclease after its binding to electrode surface. Physical adsorption method by means of electrostatic forces is the simplest strategy to immobilize the aptamer on the transducer surface. Aptamer immobilization methods generally operate through chemisorption of thiol onto gold, followed by formation of self assembled monolayer (SAMs) by the attachment of amine-terminated aptamer to a thiol end group. Similarly, thiol-tethered aptamers have been reported for planar gold (Baker et al., 2006; Li et al., 2008). This method, however, has the disadvantage of low stability caused by desorption from the surface. The use of gold nanoparticles (NPs) as immobilization support for aptamer to fabricate aptasensors has also been very well established. The immobilization of aptamer on gold NPs has been performed through direct attachment on gold using a symmetric mixed disulfide (Liu and Lu, 2004). The other used format is a complex dipstick in which two different types of gold NPs are used. Metal NPs have also been used for the immobilization of DNA and could be a useful alternative to gold NPs in future (Wu et al., 2007).

To perform covalent attachment to chemically modified surface, aptamers labeled with chemical functional groups interact with the corresponding chemical groups to form a layer of ordered film of aptamer on the sensor surface (Lee et al., 2009; Actis et al., 2011). The most commonly used groups for surface attachment are hydroxyl, amine, and carboxylic acid surface functional groups. Silicates and silicones have also been used as immobilization support for the fabrication of aptasensors (Zhu et al., 2006). The interaction between avidin (or streptavidin) and biotin has been exploited for surface immobilization of number of biorecognition elements, including aptamer (Becker and Wilchek, 1972). Avidin or streptavidin can easily be immobilized on the electrode surface either through physical adsorption or chemical bonding. The immobilization of aptamers on the transducer surface by hybridization with partially complementary oligonucleotide is another fixation approach. Oligonucleotides complementary to aptamers are immobilized on the electrode surfaces. However, due to the involvement of annealing and hybridization, it is difficult to control the experimental conditions (Zhang et al., 2007; Han et al., 2009). Electrochemical aptasensors have also been fabricated by incorporating aptamers into single walled carbon nanotubes (So et al., 2005). Similarly, multiwalled carbon nanotubes have been used as modifier to immobilize amine linked aptamer on the screen printed carbon electrodes.

## Electrochemical aptasensor for environmental monitoring

**Figure 4:**
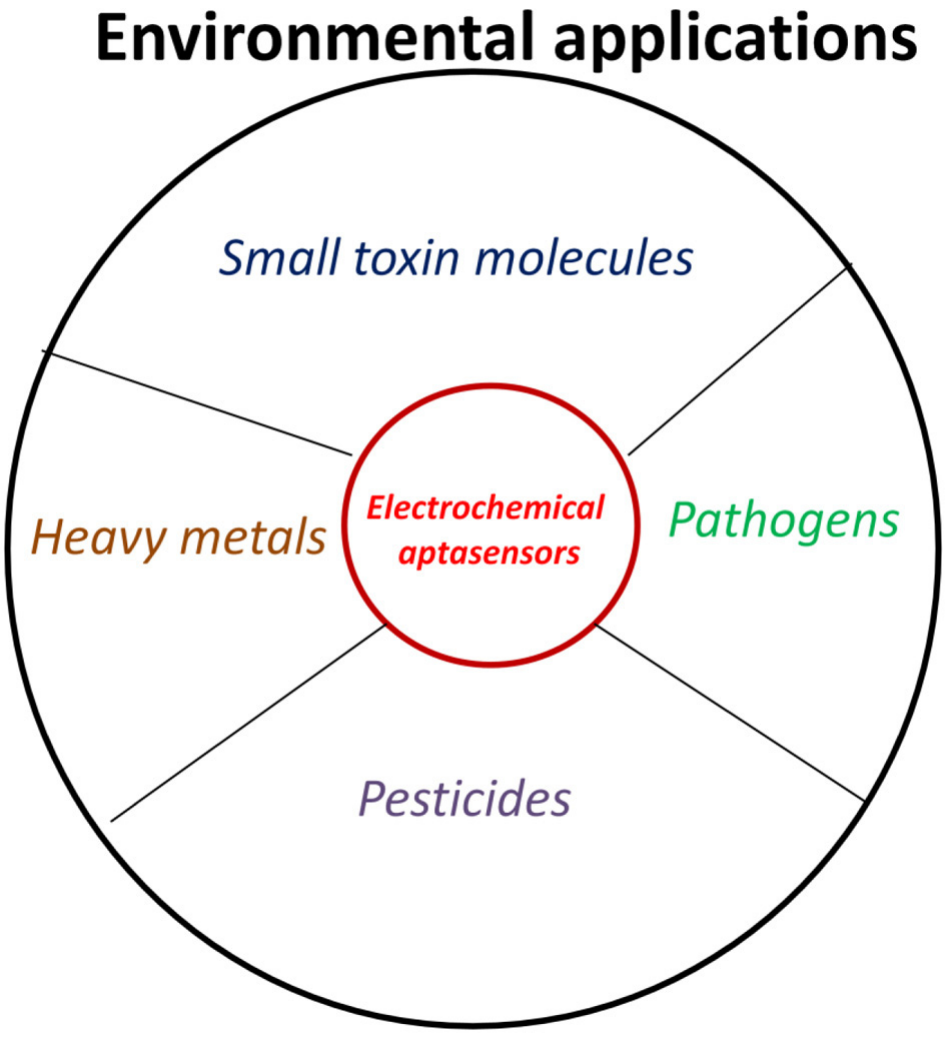
**Environmental applications of electrochemical aptasensors**.

Environment can be contaminated by several chemical compounds, toxins, pathogens… etc. resulting in different types of environmental borne diseases. As an example, according to World Health Organization (WHO), food borne and waterborne diarrheal diseases results in 2.2 million deaths per annum, and out of which 1.9 million are children (http://www.who.int/foodsafety/en/). The increasing public health problems and concerns and subsequently their consequences demands the design and fabrication of more reliable and field suitable technologies for on site and cost effective monitoring of environmental contaminants (Sett et al., 2012). In this context, electrochemical aptansensors have been emerged as potential candidate in monitoring environmental contaminants (Figure 4, Table 1). Electrochemical aptasensor have the potential to address many of the challenges associated with the traditional methods with similar or improved specificity and affinity characteristics in comparison to antibody based assays/sensors. Based on the nature of the target analyte, electrochemical aptasensor designed for environmental monitoring are classified into two major groups, for simplicity and better understanding of the scientific community.

**Table 1:** **Electrochemical aptasensors designed for the detection of different target analytes**.

**Sr no**	**Analyte**	**Transduction methodology**	**Year**	**References**
1	Theophylline	RNA aptamer-based biosensor	2008	Ferapontova et al., 2008
2	Cocaine	Aptameric biosensor	2008	Chen et al., 2008
3	Chloramphenicol	RNA aptamers	1997	Burke et al., 1997
4	Chloramphenicol	DNA aptamers	2011	Mehta et al., 2011
5	Tetracycline	RNA aptamer	2001	Berens et al., 2001
6	Tetracycline	Electrochemical aptasensor	2010	Kim et al., 2010
7	Tetracycline	Aptamer biosensor	2010	Zhang et al., 2010
8	Tetracycline	Aptamer-based assay	2012	Jeong and Rhee Paeng, 2012
9	Mycotoxins	DNA aptamer	2008	Cruz-Aguado and Penner, 2008
10	Endotoxin	Electrochemical aptasensor	2012	Kim et al., 2012
11	Bisphenol a	DNA aptamers	2011	Jo et al., 2011
12	Acetamiprid	DNA aptamer	2011	He et al., 2011
13	Pesticides	DNA aptamers	2012	Wang et al., 2012
14	Virus-infected cells	Aptamers	2009	Tang et al., 2009
15	*Escherichia coli*	Aptamer-functionalized carbon-nanotube	2008	So et al., 2008
16	Anthrax	Nano aptasensor	2010	Cella et al., 2010

1. Electrochemical aptasensor for small molecules detection.
2. Electrochemical aptasensor for pathogen detection.

### Electrochemical aptasensors against small molecules

Small target analytes including antibiotics, toxins, pesticides and heavy metals can be present in a variety of environmental samples. One such example is theophyline, commonly used bronchodilator used for asthma patients, however, its overdose lead to sever toxicity like seizure.. etc. For this molecule, RNA based electrochemical aptasensors have been reported in the literature to monitor its level in human serum (Ferapontova et al., 2008). Similarly, aptamers against drugs such as cocaine have been developed and successfully implemented for their detection (Chen et al., 2008).

#### Electrochemical aptasensors against antibiotics

Antibiotics are used to farm animal along with their feed for prophylactic and therapeutic purpose. However, a certain amount of these antibiotics remains un metabolized and accumulate in the tissue or excrete in the surroundings (Chen et al., 2008). The presence of antibiotics in the environment may results in antibiotic resistance with the subsequent possibility of transmittance to the human being through food chain. Chloramphenicol, antimicobal drug, has lost its favor due to resistance and serious side effects like aplastic anemia. Burk et al. were the first one who designed and reported an aptamer against chloramphenicol (Burke et al., 1997). However, RNA aptamers are suspected to nuclease attack and needs transcription and reverse transcription, making it difficult to screen. Recently, Metha et al. developed DNA aptamers, and the designed aptamers were used for the electrochemical detection of chloramphenicol. The aptamers were immobilized onto gold electrode surface via self-assembly approach. The developed aptasensors were very sensitive and selective toward detection of chloramphenicol (Mehta et al., 2011). Tetracyclines are another group of broad spectrum antibiotics which inhibits prokaryotic translation (Spahn and Prescott, 1996). They are often used as veterinary drug to promote growth in animals. Their residues have been detected in meat, milk, honey, eggs etc. (Pena et al., 1999; Muriuki et al., 2001). On the other hand, this antibiotic has also been reported as hepatotoxic to pregnant women (Gwee, 1982). Traditional detection methods such as HPLC have been failed for their detection due to lack of specificity. Recently, aptamer have been selected for the detection of tetracyclines (Berens et al., 2001). In this context, Kim et al. recently designed an electrochemical aptasensor over glassy carbon electrodes (Kim et al., 2010). After this work, many electrochemical aptasensors were designed and reported in the literature for the detection of tetracyclines (Kim et al., 2010; Zhang et al., 2010; Jeong and Rhee Paeng, 2012). Xiao et al. improved the structure of the aptamer to obtain a very high affinity constant against tetracyclines (Kd ~ 0.8 nM) (Xiao et al., 2008). Zhang et al. reported a rapid electrochemical aptasensor using aptamers immobilized over glassy carbon electrodes. The aptasensor was able to rapidly detect tetracycline in milk with very high sensitivity (Zhang et al., 2010). Similarly, a competitive enzyme linked aptamers assay (ELIAA) for tetracycline was developed by Jheong et al. using both DNA and RNA aptamers (Jeong and Rhee Paeng, 2012). Zhou et al. fabricated a simple electrochemical tetracycline aptasensor with multi-walled carbon nanotubes modification. The electrochemical aptasensor exhibited a good sensitivity and was successfully applied to the determination of tetracyclines in real samples (Zhou et al., 2012).

#### Electrochemical aptasensors for toxins

Mycotoxins are the major group of toxins that are present in our food. They are group of naturally occurring chemicals produced by molds growing on different typed of crops. They have several adverse impacts on human health such as gastrointestinal diseases to kidney damage and immune suppression. Among the mycotoxins, Ochratoxin A (OTA) is the most commonly occurring toxins, and a DNA aptamer was selected against it in 2008 (Cruz-Aguado and Penner, 2008). Afterwards, numerous number of electrochemical aptasensors were developed to detect this toxins (Rhouati et al., 2013b). Bacterial endotoxins are also one of the major contaminants present in pharmaceutical products, causing sever septic shock in humans and animals (Magalhaes et al., 2007). Kim et al. fabricated an electrochemical aptasensors for the detection of endotoxins from crude biological liquor (Kim et al., 2012). Seeds of the leguminous herb lupin are the widely used source of low cost protein. However, there have been an increasing number of cases reporting severe allergic reactions to these seeds. To meet this challenge and detect Lupin allergen levels in food, a DNA aptamer based assay was developed by Nadal et al. (2012). Many toxins are excreted by human sand animals and subsequently entering into water effluents. Endocrine disrupting compounds form a major class of pollutants with several health hazards (Chang et al., 2009). Estradiol is one such compound which has adverse effects on the male reproductive system. Recently, Yildrim et al. designed a DNA sensor based on fluorescence detection method for environmental water samples (Yildirim et al., 2012). However, this method can be adapted to the electrochemical methods for better analytical performance in future. Bisphenol A is used as a monomer constituent in plastic poly hydrocarbonate products. However, it has potential harmful effect for humans and animals as it interact with endocrine system by blocking of estrogen with its receptors. Jo et al. developed a sol gel biochip aptasensor to detect Bisphenol A in water samples. Further, electrochemical aptasensors can be designed to measure Bisphenol A based on the aptamer platform (Jo et al., 2011). Zhou et al. developed a simple and label-free electrochemical aptasensor for bisphenol A (BPA) determination. The method was based on the gold nanoparticles dotted graphene nanocomposite film modified glassy carbon electrode. The proposed aptasensor was rapid, convenient and low-cost for effective sensing of BPA (Zhou et al., 2014).

#### Electrochemical aptasensors against heavy metals

Heavy metals including mercury, arsenic.. etc. cause severe toxicity to nervous and endocrine system, and also heart problems, skin lesions, and cancer. According to official sources, arsenic level in water is exceeded than the allowable limit leading to serve arsenic toxicity among inhabitants. As a result, an aptansensor has been proposed by Kim et al. group to detect arsenic, with potential tool to be used as alarming method (Kim et al., 2009). Mercury has also been detected by the combination of gold nanoparticles and aptamer with optical output (Li et al., 2009; Helwa et al., 2012). However, the mercury specific aptamer can be very easily extended to electrochemical sensing platform in future. Li et al. developed an electrochemical DNAzyme sensor for sensitive and selective detection of lead ion (Pb2+), taking advantage of catalytic reactions of a DNAzyme upon its binding to Pb2+ and the use of DNA-Au bio-bar codes to achieve signal enhancement. Although this DNAzyme sensor was demonstrated for the detection of Pb2+, it has the potential to serve as a general platform for design sensors for other small molecules and heavy metal ions (Shen et al., 2008). Chen et al. reported a highly sensitive and specific electrochemical aptasensor for Cu2+ detection based on gold nanoparticles. Rapidity, simplicity, and excellent selectivity made it suitable for practical use in determination of Cu2+ from lake samples (Chen et al., 2011).

Till date, a number of aptamer based sensors have been developed for environmental applications to detect very low level of heavy metals. But still there are many toxic heavy metals for which aptamer have not been designed, so future work may focus to synthesize aptamer against the rest of toxin metal target analytes.

#### Electrochemical aptasensor for pesticides

Atrazine is one of the most intensively used pesticides to inhibit the growth of weeds. However, its presence may cause reproductive damage in humans. Sinha et al. screened a series of aptamers against atrazine and then cloned them into *E. coli* cells (Sinha et al., 2010). The screened aptamers may find potential applications in developing electrochemical aptasensors to detect atrazine in future. Similarly, insecticides are also applied to crops to protect them from insect attack. One such example is acetamipride, which when leached into the environment can cause toxicity in humans and animals. A series of aptamers which bind to acetamipirid with high dissociation constant have been synthesized recently (He et al., 2011; Wang et al., 2012). Wang et al. designed a DNA aptamer which was able to detect up to four highly poisonous organo-phosphorous pesticides including phorate, profenofos, isocarbophos, and omethoateas (Shen et al., 2008). Fan et al. developed an aptasensor for sensitive and selective detection of acetamiprid based on electrochemical impedance spectroscopy (Fan et al., 2013). To improve sensitivity of the aptasensor, gold nanoparticles were electrodeposited on the bare gold electrode surface by cycle voltammetry. The applicability of the developed aptasensor was successfully evaluated by determining acetamiprid in the real samples, wastewater and tomatoes. Although aptamers have been selected against various types of pesticides, however, their potential as ligand molecules in a biosensor is mainly unexplored.

### Electrochemical aptasensors against pathogens

Detection, identification, and quantification of microbial pathogens are crucial for public health protection. Areas where detection of microbial pathogens is critical include water and environmental analysis. Aptasensors capable of rapidly detecting pathogens with improved analytical performance are highly desired to meet the increasing environmental problems. The aptamer based assays are able to identify and detect different types of pathogens without prior information of their membrane molecules and structural genes. Among the pathogens, aptamers assays against virus are in their infancy and some of the reported aptamers against specific viruses could get potential in the fabrication of biosensors. Various aptasensors platform have been reported in the literature for various types of viruses (Minunni et al., 2004; Tombelli et al., 2005; Lee et al., 2007; Tang et al., 2009). Similarly, detection of bacteria is also a relatively emerging and new area. Aptasensors based on different types of nanomaterials in combination with electrochemical transduction output are described for the measurement of bacteria (So et al., 2008; Cella et al., 2010). Aptamer-functionalized single walled carbon nanotube field-effect transistor (SWNTFET) arrays aptasensor was developed to detect *E. coli* DH5 α (Fan et al., 2013). The binding between *E. coli* cells and the aptamer-functionalized FET resulted in a change in conductance of the samples which was subsequently related to detection mechanism. Recently, bioterrorism has been considered as a major threat to national security. One of the leading examples is Anthrax that is associated with the spores of gram positive bacteria Bacillus anthracis. Cella et al. reported an aptasensor for detection of protective antigen of anthrax (Tang et al., 2009). The aptasensor consisted of single stranded DNA aptamer functionalized to single walled carbon nanotubes having sensitivity in the nanomolar range.

## Conclusion and perspectives

Despite of the attractive advantages, aptamer based assays are still under phase of development as compared to immunoassays for environmental monitoring. The primary hurdles are the limited number of available aptamers and relatively poor knowledge of aptamer immobilization strategies. However, recent years have witnessed important and rapid advances in the sequences of new aptamers, along with integration of new nanomaterials in aptamer based assays, rapidly improving the existing procedures. Electrochemical aptasensors reveal certain advantages when compared to the optical sensors, For example, the possibility of integrating signal amplifying catalytic or biocatalytic labels to the aptamer-target complex enables the highly sensitive detection of the target substrate. Recent advances have also witnessed the electrochemical label free detection, thus overcoming the requirement of labels. Even though substantial progress has been accomplished in the design of electrochemical aptasensors, several exciting, and improving opportunities still exist in the field of aptasensors. For example, the use of synthetically modified nucleotide as co-component and integration of the binding properties of aptamer with DNAzyme may yield the hybrid structures with superior sensing functions by combining selected binding and catalytic properties of aptamer. Overall, the potential of electrochemical aptasensors is immense in the environmental monitoring, and this exciting and challenging area is on the brink of exponential growth. The future development in aptasensors with respect to environmental monitoring may focus on the design of aptamers against unexplored target analyte, and then their subsequent integration in the electrochemical platform to monitor those target analytes.

### Conflict of interest statement

The authors declare that the research was conducted in the absence of any commercial or financial relationships that could be construed as a potential conflict of interest.

## References

[B1] Actis P. Rogers A. Nivala J. Vilozny B. Seger R. A. Jejelowo O. (2011). Reversible thrombin detection by aptamer functionalized STING sensors. Biosens. Bioelectron. 26, 4503–4507 10.1016/j.bios.2011.05.010 21636261 PMC3120900

[B2] Baker B. R. Lai R. Y. Wood M. S. Doctor E. H. Heeger A. J. Plaxco K. W. (2006). An electronic, aptamer-based small-molecule sensor for the rapid, label-free detection of cocaine in adulterated samples and biological fluids. J. Am. Chem. Soc. 128, 3138–3139 10.1021/ja056957p 16522082

[B3] Barthelmebs L. Hayat A. Limiadi A. W. Marty J. L. Noguer T. (2011). Electrochemical DNA aptamer-based biosensor for OTA detection, using superparamagnetic nanoparticles. Sens. Actuat. B Chem. 156, 932–937 10.1016/j.snb.2011.03.00821256729

[B4] Becker J. M. Wilchek M. (1972). Inactivation by avidin of biotin-modified bacteriophage. Biochim. Biophys. Acta 264, 165–170 10.1016/0304-4165(72)90127-4 4553809

[B5] Berens C. Thain A. Schroeder R. (2001). A tetracycline-binding RNA aptamer. Bioorg. Med. Chem. 9, 2549–2556 10.1016/S0968-0896(01)00063-3 11557342

[B6] Burke D. H. Hoffman D. C. Brown A. Hansen M. Pardi A. Gold L. (1997). RNA aptamers to the peptidyl transferase inhibitor chloramphenicol. Chem. Biol. 4, 833–843 10.1016/S1074-5521(97)90116-2 9384530

[B7] Castillo G. Lamberti I. Mosiello L. Hianik T. (2012). Impedimetric DNA aptasensor for sensitive detection of ochratoxin A in food. Electroanalysis 24, 512–520 10.1002/elan.20110048521514815

[B8] Cella L. N. Chen W. Mulchandani A. (2011). Chapter 4 aptamer-based biosensor for environmental monitoring, in Nucleic Acid Biosensors for Environmental Pollution Monitoring, eds Mascini M. Palchetti I. (The Royal Society of Chemistry), 61–81 10.1039/9781849732697-00061

[B9] Cella L. N. Sanchez P. Zhong W. Myung N. V. Chen W. Mulchandani A. (2010). Nano aptasensor for protective antigen toxin of anthrax. Anal. Chem. 82, 2042–2047 10.1021/ac902791q 20136122 PMC2930939

[B10] Centi S. Tombelli S. Minunni M. Mascini M. (2007). Aptamer-based detection of plasma proteins by an electrochemical assay coupled to magnetic beads. Anal. Chem. 79, 1466–1473 10.1021/ac061879p 17297945

[B11] Chakraborty B. Jiang Z. Li Y. Yu H.-Z. (2009). Rational design and performance testing of aptamer-based electrochemical biosensors for adenosine. J. Electroanal. Chem. 635, 75–82 10.1016/j.jelechem.2009.08.006

[B12] Chang H. S. Choo K. H. Lee B. Choi S. J. (2009). The methods of identification, analysis, and removal of endocrine disrupting compounds (EDCs) in water. J. Hazard. Mater. 172, 1–12 10.1016/j.jhazmat.2009.06.135 19632774

[B13] Chen J. Jiang J. Gao X. Liu G. Shen G. Yu R. (2008). A new aptameric biosensor for cocaine based on surface-enhanced raman scattering spectroscopy. Chemistry 14, 8374–8382 10.1002/chem.200701307 18666292

[B14] Chen Z. Li L. Mu X. Zhao H. Guo L. (2011). Electrochemical aptasensor for detection of copper based on a reagentless signal-on architecture and amplification by gold nanoparticles. Talanta 85, 730–735 10.1016/j.talanta.2011.04.056 21645766

[B15] Cheng A. K. H. Ge B. Yu H. Z. (2007). Aptamer-based biosensors for label-free voltammetric detection of lysozyme. Anal. Chem. 79, 5158–5164 10.1021/ac062214q 17566977

[B16] Cruz-Aguado J. A. Penner G. (2008). Determination of ochratoxin a with a DNA aptamer. J. Agric. Food Chem. 56, 10456–10461 10.1021/jf801957h 18983163

[B17] Ellington A. D. Szostak J. W. (1990). *In vitro* selection of RNA molecules that bind specific ligands. Nature 346, 818–822 10.1038/346818a0 1697402

[B18] Fan L. Zhao G. Shi H. Liu M. Li Z. (2013). A highly selective electrochemical impedance spectroscopy-based aptasensor for sensitive detection of acetamiprid. Biosens. Bioelectron. 43, 12–18 10.1016/j.bios.2012.11.033 23274191

[B19] Ferapontova E. E. Olsen E. M. Gothelf K. V. (2008). An RNA aptamer-based electrochemical biosensor for detection of theophylline in serum. J. Am. Chem. Soc. 130, 4256–4258 10.1021/ja711326b 18324816

[B20] Gorodetsky A. A. Buzzeo M. C. Barton J. K. (2008). DNA-mediated electrochemistry. Bioconjugate Chem. 19, 2285–2296 10.1021/bc8003149 18980370 PMC2663395

[B21] Gwee M. C. E. (1982). Can tetracycline-induced fatty liver in pregnancy be attributed to choline deficiency? Med. Hypotheses 9, 157–162 10.1016/0306-9877(82)90131-1 6183565

[B22] Hamula C. L. A. Guthrie J. W. Zhang H. Li X.-F. Le X. C. (2006). Selection and analytical applications of aptamers. Trends Anal. Chem. 25, 681–691 10.1016/j.trac.2006.05.007

[B23] Han K. Chen L. Lin Z. Li G. (2009). Target induced dissociation (TID) strategy for the development of electrochemical aptamer-based biosensor. Electrochem. Commun. 11, 157–160 10.1016/j.elecom.2008.10.054

[B24] Hayat A. Andreescu S. (2013). Nanoceria Particles as catalytic amplifiers for alkaline phosphatase assays. Anal. Chem. 85, 10028–10032 10.1021/ac4020963 24053108

[B25] Hayat A. Andreescu S. Marty J.-L. (2013a). Design of PEG-aptamer two piece macromolecules as convenient and integrated sensing platform: application to the label free detection of small size molecules. Biosens. Bioelectron. 45, 168–173 10.1016/j.bios.2013.01.059 23500359

[B26] Hayat A. Barthelmebs L. Sassolas A. Marty J. L. (2011). An electrochemical immunosensor based on covalent immobilization of okadaic acid onto screen printed carbon electrode via diazotization-coupling reaction. Talanta 85, 513–518 10.1016/j.talanta.2011.04.034 21645734

[B27] Hayat A. Barthelmebs L. Sassolas A. Marty J. L. (2012a). Development of a novel label-free amperometric immunosensor for the detection of okadaic acid. Anal. Chim. Acta. 724, 92–97 10.1016/j.aca.2012.02.035 22483215

[B28] Hayat A. Haider W. Rolland M. Marty J.-L. (2013b). Electrochemical grafting of long spacer arms of hexamethyldiamine on a screen printed carbon electrode surface: application in target induced ochratoxin A electrochemical aptasensor. Analyst 138, 2951–2957 10.1039/c3an00158j 23535890

[B29] Hayat A. Paniel N. Rhouati A. Marty J. L. Barthelmebs L. (2012b). Recent advances in ochratoxin A-producing fungi detection based on PCR methods and ochratoxin A analysis in food matrices. Food Control 26, 401–415 10.1016/j.foodcont.2012.01.060

[B30] Hayat A. Yang C. Rhouati A. Marty J. (2013c). Recent advances and achievements in nanomaterial-based, and structure switchable aptasensing platforms for ochratoxin A detection. Sensors 13, 15187–15208 10.3390/s131115187 24201319 PMC3871093

[B31] He J. Liu Y. Fan M. Liu X. (2011). Isolation and identification of the DNA aptamer target to acetamiprid. J. Agric. Food Chem. 59, 1582–1586 10.1021/jf104189g 21306108

[B32] Helwa Y. Dave N. Froidevaux R. Samadi A. Liu J. (2012). Aptamer-functionalized hydrogel microparticles for fast visual detection of mercury(II) and adenosine. ACS Appl. Mat. Interfaces 4, 2228–2233 10.1021/am300241j 22468717

[B33] Hermann T. Patel D. J. (2000). Adaptive recognition by nucleic acid aptamers. Science 287, 820–825 10.1126/science.287.5454.820 10657289

[B34] Ihara T. Maruo Y. Takenaka S. Takagi M. (1996). Ferrocene-oligonucleotide conjugates for electrochemical probing of DNA. Nucleic Acids Res. 24, 4273–4280 10.1093/nar/24.21.4273 8932383 PMC146233

[B35] Ikebukuro K. Kiyohara C. Sode K. (2005). Novel electrochemical sensor system for protein using the aptamers in sandwich manner. Biosens. Bioelectron. 20, 2168–2172 10.1016/j.bios.2004.09.002 15741093

[B36] Jenison R. D. Gill S. C. Pardi A. Polisky B. (1994). High-resolution molecular discrimination by RNA. Science 263, 1425–1429 10.1126/science.7510417 7510417

[B37] Jeong S. Rhee Paeng I. (2012). Sensitivity and selectivity on aptamer-based assay: the determination of tetracycline residue in bovine milk. ScientificWorldJournal 2012:159456 10.1100/2012/159456 22547977 PMC3324139

[B38] Jo M. Ahn J. Y. Lee J. Lee S. Hong S. W. Yoo J. W. (2011). Development of single-stranded DNA aptamers for specific Bisphenol a detection. Oligonucleotides 21, 85–91 10.1089/oli.2010.0267 21413891 PMC3125561

[B39] Kelley S. O. Barton J. K. Jackson N. M. Hill M. G. (1997). Electrochemistry of methylene blue bound to a DNA-modified electrode. Bioconjug. Chem. 8, 31–37 10.1021/bc960070o 9026032

[B40] Kelley S. O. Jackson N. M. Hill M. G. Barton J. K. (1999). Long-range electron transfer through DNA films. Angew. Chem. Int. Ed. 38, 941–945 29711858 10.1002/(SICI)1521-3773(19990401)38:7<941::AID-ANIE941>3.0.CO;2-7

[B41] Kim M. Um H.-J. Bang S. Lee S.-H. Oh S.-J. Han J.-H. (2009). Arsenic removal from vietnamese groundwater using the arsenic-binding DNA aptamer. Environ. Sci. Technol. 43, 9335–9340 10.1021/es902407g 20000526

[B42] Kim S. E. Su W. Cho M. Lee Y. Choe W. S. (2012). Harnessing aptamers for electrochemical detection of endotoxin. Anal. Biochem. 424, 12–20 10.1016/j.ab.2012.02.016 22370280

[B43] Kim Y. J. Kim Y. S. Niazi J. H. Gu M. B. (2010). Electrochemical aptasensor for tetracycline detection. Bioprocess Biosyst. Eng. 33, 31–37 10.1007/s00449-009-0371-4 19701778

[B44] Lai R. Y. Seferos D. S. Heeger A. J. Bazan G. C. Plaxco K. W. (2006). Comparison of the signaling and stability of electrochemical DNA sensors fabricated from 6- or 11-carbon self-assembled monolayers. Langmuir 22, 10796–10800 10.1021/la0611817 17129062

[B45] Lee H.-S. Kim K. S. Kim C.-J. Hahn S. K. Jo M.-H. (2009). Electrical detection of VEGFs for cancer diagnoses using anti-vascular endotherial growth factor aptamer-modified Si nanowire FETs. Biosens. Bioelectron. 24, 1801–1805 10.1016/j.bios.2008.08.036 18835770

[B46] Lee S. Kim Y. S. Jo M. Jin M. Lee D.-K. Kim S. (2007). Chip-based detection of hepatitis C virus using RNA aptamers that specifically bind to HCV core antigen. Biochem. Biophys. Res. Commun. 358, 47–52 10.1016/j.bbrc.2007.04.057 17475212

[B47] Li B. Wang Y. Wei H. Dong S. (2008). Amplified electrochemical aptasensor taking AuNPs based sandwich sensing platform as a model. Biosens. Bioelectron. 23, 965–970 10.1016/j.bios.2007.09.019 17997091

[B48] Li L. Li B. Qi Y. Jin Y. (2009). Label-free aptamer-based colorimetric detection of mercury ions in aqueous media using unmodified gold nanoparticles as colorimetric probe. Anal. Bioanal.Chem. 393, 2051–2057 10.1007/s00216-009-2640-0 19198811

[B49] Liu J. Lu Y. (2004). Adenosine-dependent assembly of aptazyme-functionalized gold nanoparticles and its application as a colorimetric biosensor. Anal. Chem. 76, 1627–1632 10.1021/ac0351769 15018560

[B50] Lu Y. Li X. Zhang L. Yu P. Su L. Mao L. (2008). Aptamer-based electrochemical sensors with aptamer-complementary DNA oligonucleotides as probe. Anal. Chem. 80, 1883–1890 10.1021/ac7018014 18290636

[B51] Magalhaes P. O. Lopes A. M. Mazzola P. G. Rangel-Yagui C. Penna T. C. Pessoa A. Jr. (2007). Methods of endotoxin removal from biological preparations: a review. J. Pharm. Pharm. Sci. 10, 388–404 17727802

[B52] Mehta J. Van Dorst B. Rouah-Martin E. Herrebout W. Scippo M. L. Blust R. (2011). *In vitro* selection and characterization of DNA aptamers recognizing chloramphenicol. J. Biotechnol. 155, 361–369 10.1016/j.jbiotec.2011.06.043 21839787

[B53] Minunni M. Tombelli S. Gullotto A. Luzi E. Mascini M. (2004). Development of biosensors with aptamers as bio-recognition element: the case of HIV-1 Tat protein. Biosens. Bioelectron. 20, 1149–1156 10.1016/j.bios.2004.03.037 15556361

[B54] Mir M. Vreeke M. Katakis I. (2006). Different strategies to develop an electrochemical thrombin aptasensor. Electrochem. Commun. 8, 505–511 10.1016/j.elecom.2005.12.022

[B55] Muriuki F. K. Ogara W. O. Njeruh F. M. Mitema E. S. (2001). Tetracycline residue levels in cattle meat from Nairobi salughter house in Kenya. J. Vet. Sci. 2, 97–101 14614278

[B56] Nadal P. Pinto A. Svobodova M. Canela N. O'Sullivan C. K. (2012). DNA aptamers against the Lup an 1 food allergen. PLoS ONE 7:e35253 10.1371/journal.pone.0035253 22529997 PMC3328447

[B57] O'Sullivan C. (2002). Aptasensors – the future of biosensing? Anal. Bioanal. Chem. 372, 44–48 10.1007/s00216-001-1189-3 11939212

[B58] Paniel N. Baudart J. Hayat A. Barthelmebs L. (2013). Aptasensor and genosensor methods for detection of microbes in real world samples. Methods 64, 229–240 10.1016/j.ymeth.2013.07.001 23872322

[B59] Park K. Kwon D. Kwak J. (2011). Aptamer based electrochemical sensor system for protein using the generation/collection mode of scanning electrochemical microscope (SECM). J. Nanosci. Nanotechnol. 11, 4305–4311 10.1166/jnn.2011.3671 21780447

[B60] Pena A. L. Lino C. M. Silveira I. N. (1999). Determination of oxytetracycline, tetracycline, and chlortetracycline in milk by liquid chromatography with postcolumn derivatization and fluorescence detection. J. AOAC Int. 82, 55–60 10028670

[B61] Pheeney C. G. Barton J. K. (2012). DNA electrochemistry with tethered methylene blue. Langmuir 28, 7063–7070 10.1021/la300566x 22512327 PMC3398613

[B62] Radi A. E. Sanchez J. L. A. Baldrich E. O'Sullivan C. K. (2006). Reagentless, reusable, ultrasensitive electrochemical molecular beacon aptasensor. J. Am. Chem. Soc. 128, 117–124 10.1021/ja053121d 16390138

[B63] Rhouati A. Hayat A. Hernandez D. B. Meraihi Z. Munoz R. Marty J. L. (2013a). Development of an automated flow-based electrochemical aptasensor for on-line detection of Ochratoxin A. Sens. Actuat. B Chem. 176, 1160–1166 10.1016/j.snb.2012.09.111

[B64] Rhouati A. Yang C. Hayat A. Marty J.-L. (2013b). Aptamers: a promising tool for ochratoxin a detection in food analysis. Toxins 5, 1988–2008 10.3390/toxins5111988 24196457 PMC3847711

[B65] Seeman N. C. (2003). Biochemistry and structural DNA nanotechnology: an evolving symbiotic relationship†. Biochemistry 42, 7259–7269 10.1021/bi030079v 12809482

[B66] Seeman N. C. (2010). Nanomaterials based on DNA. Annu. Rev. Biochem. 79, 65–87 10.1146/annurev-biochem-060308-102244 20222824 PMC3454582

[B67] Sett A. Das S. Sharma P. Bora U. (2012). Aptasensors in health, environment and food safety monitoring. Open J. Appl. Biosens. 1, 9–19 10.4236/ojab.2012.12002

[B68] Shen L. Chen Z. Li Y. He S. Xie S. Xu X. (2008). Electrochemical DNAzyme sensor for lead based on amplification of DNA-Au bio-bar codes. Anal. Chem. 80, 6323–6328 10.1021/ac800601y 18627134

[B69] Sinha J. Reyes S. J. Gallivan J. P. (2010). Reprogramming bacteria to seek and destroy an herbicide. Nat. Chem. Biol. 6, 464–470 10.1038/nchembio.369 20453864 PMC2873063

[B70] So H.-M. Park D.-W. Jeon E.-K. Kim Y.-H. Kim B. S. Lee C.-K. (2008). Detection and titer estimation of *Escherichia coli* using aptamer-functionalized single-walled carbon-nanotube field-effect transistors. Small 4, 197–201 10.1002/smll.200700664 18214875

[B71] So H.-M. Won K. Kim Y. H. Kim B.-K. Ryu B. H. Na P. S. (2005). Single-walled carbon nanotube biosensors using aptamers as molecular recognition elements. J. Am. Chem. Soc. 127, 11906–11907 10.1021/ja053094r 16117506

[B72] Spahn C. M. Prescott C. D. (1996). Throwing a spanner in the works: antibiotics and the translation apparatus. J. Mol. Med. (Berl.) 74, 423–439 10.1007/BF00217518 8872856

[B73] Tan W. Donovan M. J. Jiang J. (2013). Aptamers from cell-based selection for bioanalytical applications. Chem. Rev. 113, 2842–2862 10.1021/cr300468w 23509854 PMC5519293

[B74] Tang Y. Ge B. Sen D. Yu H. Z. (2014). Functional DNA switches: rational design and electrochemical signaling. Chem. Soc. Rev. 43, 518–529 10.1039/c3cs60264h 24169924

[B75] Tang Z. Parekh P. Turner P. Moyer R. W. Tan W. (2009). Generating aptamers for recognition of virus-infected cells. Clin. Chem. 55, 813–822 10.1373/clinchem.2008.113514 19246617 PMC3869972

[B76] Tombelli S. Minunni M. Luzi E. Mascini M. (2005). Aptamer-based biosensors for the detection of HIV-1 Tat protein. Bioelectrochemistry 67, 135–141 10.1016/j.bioelechem.2004.04.011 16027048

[B77] Wang H.-Q. Wu Z. Tang L.-J. Yu R.-Q. Jiang J.-H. (2011). Fluorescence protection assay: a novel homogeneous assay platform toward development of aptamer sensors for protein detection. Nucleic Acids Res. 39, e122 10.1093/nar/gkr559 21742759 PMC3185441

[B78] Wang L. Liu X. Zhang Q. Zhang C. Liu Y. Tu K. (2012). Selection of DNA aptamers that bind to four organophosphorus pesticides. Biotechnol. Lett 34, 869–874 10.1007/s10529-012-0850-6 22261866

[B79] White R. J. Phares N. Lubin A. A. Xiao Y. Plaxco K. W. (2008). Optimization of electrochemical aptamer-based sensors via optimization of probe packing density and surface chemistry. Langmuir 24, 10513–10518 10.1021/la800801v 18690727 PMC2674396

[B80] Willner I. Zayats M. (2007). Electronic aptamer-based sensors. Angew. Chem. Int. Ed. Engl. 46, 6408–6418 10.1002/anie.200604524 17600802

[B81] Wu Z. S. Guo M. M. Zhang S. B. Chen C. R. Jiang J. H. Shen G. L. (2007). Reusable electrochemical sensing platform for highly sensitive detection of small molecules based on structure-switching signaling aptamers. Anal. Chem. 79, 2933–2939 10.1021/ac0622936 17338505

[B82] Xiao H. Edwards T. E. Ferré-D'Amaré A. R. (2008). Structural basis for specific, high-affinity tetracycline binding by an *in vitro* evolved aptamer and artificial riboswitch. Chem. Biol. 15, 1125–1137 10.1016/j.chembiol.2008.09.004 18940672 PMC2626642

[B83] Xiao Y. Lubin A. A. Heeger A. J. Plaxco K. W. (2005). Label-free electronic detection of thrombin in blood serum by using an aptamer-based sensor. Angew. Chem. Int. Ed. 44, 5456–5459 10.1002/anie.200500989 16044476

[B84] Yildirim N. Long F. Gao C. He M. Shi H. C. Gu A. Z. (2012). Aptamer-based optical biosensor for rapid and sensitive detection of 17beta-estradiol in water samples. Environ. Sci. Technol. 46, 3288–3294 10.1021/es203624w 22296460

[B85] Yu H.-Z. Luo C.-Y. Sankar C. G. Sen D. (2003). Voltammetric procedure for examining dna-modified surfaces: quantitation, cationic binding activity, and electron-transfer kinetics. Anal. Chem. 75, 3902–3907 10.1021/ac034318w 14572060

[B86] Zhang J. Zhang B. Wu Y. Jia S. Fan T. Zhang Z. (2010). Fast determination of the tetracyclines in milk samples by the aptamer biosensor. Analyst 135, 2706–2710 10.1039/c0an00237b 20714519

[B87] Zhang Y. L. Huang Y. Jiang J. H. Shen G. L. Yu R. Q. (2007). Electrochemical aptasensor based on proximity-dependent surface hybridization assay for single-step, reusable, sensitive protein detection. J. Am. Chem. Soc. 129, 15448–15449 10.1021/ja0773047 18031045

[B88] Zhou L. Li D.-J. Gai L. Wang J.-P. Li Y.-B. (2012). Electrochemical aptasensor for the detection of tetracycline with multi-walled carbon nanotubes amplification. Sens. Actuat. B Chem. 162, 201–208 10.1016/j.snb.2011.12.067

[B89] Zhou L. Wang J. Li D. Li Y. (2014). An electrochemical aptasensor based on gold nanoparticles dotted graphene modified glassy carbon electrode for label-free detection of bisphenol A in milk samples. Food Chem. 162, 34–40 10.1016/j.foodchem.2014.04.058 24874354

[B90] Zhu H. Suter J. D. White I. M. Fan X. (2006). Aptamer based microsphere biosensor for thrombin detection. Sensors 6, 785–795 10.3390/s6080785

[B91] Zuo X. Song S. Zhang J. Pan D. Wang L. Fan C. (2007). A target-responsive electrochemical aptamer switch (TREAS) for reagentless detection of nanomolar ATP. J. Am. Chem. Soc. 129, 1042–1043 10.1021/ja067024b 17263380

